# Chemical Exchange Saturation Transfer MR Imaging is Superior to Diffusion-Tensor Imaging in the Diagnosis and Severity Evaluation of Parkinson’s Disease: A Study on Substantia Nigra and Striatum

**DOI:** 10.3389/fnagi.2015.00198

**Published:** 2015-10-19

**Authors:** Chunmei Li, Rui Wang, Haibo Chen, Wen Su, Shuhua Li, Xuna Zhao, Jinyuan Zhou, Jian Qiao, Baohui Lou, Guodong Song, Min Chen

**Affiliations:** ^1^Department of Radiology, Beijing Hospital, Beijing, China; ^2^Department of Neurology, Beijing Hospital, Beijing, China; ^3^Department of Radiology, Johns Hopkins University, Baltimore, MD, USA; ^4^Philips Healthcare, Beijing, China

**Keywords:** chemical exchange saturation transfer, amide proton transfer, diffusion-tensor imaging, substantia nigra, striatum, Parkinson’s disease

## Abstract

Parkinson’s disease (PD) is a neurodegenerative disorder characterized by nigrostriatal cell loss. To date, the diagnosis of PD is still based primarily on the clinical manifestations, which may be typical and obvious only in advanced-stage PD. Thus, it is crucial to find a reliable marker for the diagnosis of PD. We conducted this study to assess the diagnostic efficiency of chemical exchange saturation transfer (CEST) imaging and diffusion-tensor imaging (DTI) in PD at 3 T by evaluating changes on substantia nigra and striatum. Twenty-three PD patients and twenty-three age-matched normal controls were recruited. All patients and controls were imaged on a 3-T MR system, using an eight-channel head coil. CEST imaging was acquired in two transverse slices of the head, including substantia nigra and striatum. The magnetization transfer ratio asymmetry at 3.5 ppm, MTR_asym_(3.5 ppm), and the total CEST signal intensity between 0 and 4 ppm were calculated. Multi-slice DTI was acquired for all the patients and normal controls. Quantitative analysis was performed on the substantia nigra, globus pallidus, putamen, and caudate. The MTR_asym_(3.5 ppm) value, the total CEST signal intensity, and fractional anisotropy value of the substantia nigra were all significantly lower in PD patients than in normal controls (*P* = 0.003, *P* = 0.004, and *P* < 0.001, respectively). The MTR_asym_(3.5 ppm) values of the putamen and the caudate were significantly higher in PD patients than in normal controls (*P* = 0.010 and *P* = 0.009, respectively). There were no significant differences for the mean diffusivity in these four regions between PD patients and normal controls. In conclusion, CEST MR imaging provided multiple CEST image contrasts in the substantia nigra and the striatum in PD and may be superior to DTI in the diagnosis of PD.

## Introduction

Parkinson’s disease (PD) is a neurodegenerative disorder characterized by nigrostriatal cell loss, resulting in striatal dopamine deficiency (Dauer and Przedborski, 2003). Accurate diagnosis in early stage of PD and early initiation of therapy may provide benefit for the patient (Olanow et al., 2009). Unfortunately, to date, the diagnosis of PD is still based primarily on the clinical manifestations, which may be typical and obvious only in advanced-stage PD. Thus, it is crucial to find a reliable marker for the diagnosis of PD.

Several functional MR imaging have been used to assist in the PD diagnosis, including diffusion-tensor imaging (DTI). Based on the diffusivity of water molecules, which exhibit a varying degree of tissue dependent anisotropy, two primary DTI parameters, fractional anisotropy (FA) and mean diffusivity (MD) or apparent diffusion coefficient (ADC), are associated with the directionality (anisotropy) and magnitude (diffusivity) of water diffusion, respectively (Hagmann et al., 2006). DTI has typically been used to detect axon and myelin injury in white matter of PD patients (Cnyrim et al., 2014; Kamagata et al., 2014), while some studies have also focused on evaluating the abnormalities in gray matter (Prakash et al., 2012; Zhan et al., 2012; Lenfeldt et al., 2013).

Recently, a relatively new MR technique, chemical exchange saturation transfer (CEST) imaging (Zhou and van Zijl, 2006; Kogan et al., 2013; Vinogradov et al., 2013), or more specifically, amide proton transfer (APT) MR imaging (Zhou et al., 2003), has been used to assess PD (Li et al., 2014). CEST imaging is a specific type of magnetization transfer imaging [that is sensitive to solid-like proteins in tissue (van Buchem and Tofts, 2000; Henkelman et al., 2001)]. It has recently emerged as an important contrast mechanism for MRI in the field of molecular and cellular imaging. The recent study of CEST in PD focused on the gray matter of midbrain and basal ganglia. The result indicated the potential of the CEST signal as a marker of PD (Li et al., 2014).

The aim of this study was to compare the diagnostic efficiency of the DTI and CEST techniques in PD, by evaluating the changes of substantia nigra and striatum (globus pallidus, putamen, and caudate).

## Materials and Methods

### Patients and Controls

A total of 23 PD patients (13 men and 10 women; mean age, 65.5 years; range, 46–77 years; 12.2 ± 4.3 years of education) and 23 age- and education-matched normal controls (13 men and 10 women; mean age, 64.3 years; range, 50–72 years; 12.8 ± 3.6 years of education) were recruited in this study. All patients were right-handed. Informed consent was acquired from each subject prior to participation in this study. The study was approved by the local Institutional Review Board.

Parkinson’s disease diagnosis was confirmed by an experienced movement disorder specialist according to published criteria (Hughes et al., 1992, 2001). The patients fulfilled the UK Brain Bank criteria for idiopathic PD. All PD patients were assessed with the Hoehn and Yahr (H&Y) scale by the same movement disorder specialist after overnight withdrawal of antiparkinson medication for at least 12 h.

The exclusion criteria for subjects in this study were as follows: head trauma; central nervous system infection; a history of stroke; exposure to anti-dopaminergic drugs; or other neurologic or psychiatric diseases and any structural abnormalities on brain magnetic resonance images.

### MRI Acquisition

All subjects were imaged on a 3T Philips MRI system (Achieva 3.0T; Philips Medical Systems, Best, The Netherlands), with a dual-channel body coil for transmission and an eight-channel sensitivity-encoding coil for reception. Pencil beam second-order shimming was employed. The PD patients were scanned shortly after the neurological assessment. Routine images, including axial T2-weighted, T1-weighted, and fluid-attenuated inversion recovery (FLAIR), were acquired first to exclude structural abnormality.

#### CEST/APT Imaging

The CEST/APT imaging sequence was based on a pseudo-continuous wave, off-resonance RF irradiation (saturation duration = 200 ms × 4; inter-pulse delay, 10 ms; power level = 2 μT) and a single-shot, turbo-spin-echo readout. The parameters were as follows: repetition time = 3000 ms; echo time = 7.9 ms; turbo-spin-echo factor = 54; field of view = 230 mm × 221 mm; matrix = 105 × 100; and slice thickness = 6 mm. Two transverse slices of the head were acquired, including the basal ganglia and midbrain. A multi-offset, multi-acquisition CEST/APT imaging protocol, as reported before (Li et al., 2014), was used. The 31 offsets were 0, ±0.25, ±0.5, ±0.75, ±1 (2), ±1.5 (2), ±2 (2), ±2.5 (2), ±3 (2), ±3.25 (2), ±3.5 (8), ±3.75 (2), ±4 (2), ±4.5, ±5, and ±6 ppm (the values in parentheses represented the number of acquisitions, which was 1, if not specified). An unsaturated image was acquired for the signal normalization. The acquisition time was about 3 min per slice.

#### Diffusion-Tensor Imaging

The parameters were as follows: repetition time = shortest (actual TR = 5472 ms); echo time = shortest (actual TE: 93 ms); *b* values = 0, 1000 s/mm^2^; diffusion gradient directions = 31; field of view = 240 mm × 240 mm; matrix = 128 × 128; number of excitations = 1; slice thickness = 3 mm; gap = 0; and slice number = 40. The acquisition time was about 7.5 min.

### Imaging Processing

#### CEST/APT Imaging

The APT imaging analysis was performed using in-house developed software, based on the Interactive Data Language (IDL, ITT Visual Information Solutions, Boulder, CO, USA) environment. The measured magnetization transfer spectra (*S*_sat_/*S*_0_, in which *S*_sat_ and *S*_0_ are the signal intensities with and without selective RF irradiation, respectively, plotted as a function of saturation frequency offset, relative to water) were corrected for field inhomogeneity effects on a pixel-by-pixel basis. CEST imaging is usually quantified through the magnetization transfer ratio (MTR = 1 − *S*_sat_/*S*_0_) asymmetry (MTR_asym_) analysis with respect to the water resonance:

(1) $$\begin{array}{l} {\text{MTR}}_{\text{asym}}(\text{offset})=\text{MTR}(+\text{offset})-\text{MTR}(-\text{offset})\\ \;=\left[S_{\text{sat}}(-\text{offset})-S_{\text{sat}}(+\text{offset)}\right]/S_{0}. \end{array}$$

We defined the total CEST signal intensity, ${\text{MTR}}_{\text{asym}}^{\text{total}}$, as the integral of the MTR_asym_ spectrum in the range of 0–4 ppm.

Specifically for APT imaging, we have:

(2) $${\text{MTR}}_{\text{asym}}(3.5\text{ppm})=\text{APTR}+{\text{MTR}}_{\text{asym}}^{\prime }(3.5\text{ppm}),$$

where APTR is the proton transfer ratio for the amide protons associated with mobile cellular proteins and peptides in tissue. ${\text{MTR}}_{\text{asym}}^{\prime }$ was previously thought to be the inherent MTR_asym_ of the solid-phase magnetization transfer effect (Zhou et al., 2008). However, the semi-solid conventional magnetization transfer effect was thought to be symmetrical around the water resonance due to a very broad-spectrum distribution. Several recent studies suggest that ${\text{MTR}}_{\text{asym}}^{\prime }$ could be dominated by the possible intramolecular or intermolecular nuclear Overhauser enhancement effect of the upfield non-exchangeable protons (such as aliphatic protons) of mobile to relatively less mobile cellular macromolecules and metabolites (Zhou et al., 2013a; Heo et al., 2015). To account for these confounding factors, the MTR_asym_(3.5 ppm) images calculated by Eq. 2 are, in principle, APT-weighted images.

**Figure 1:**
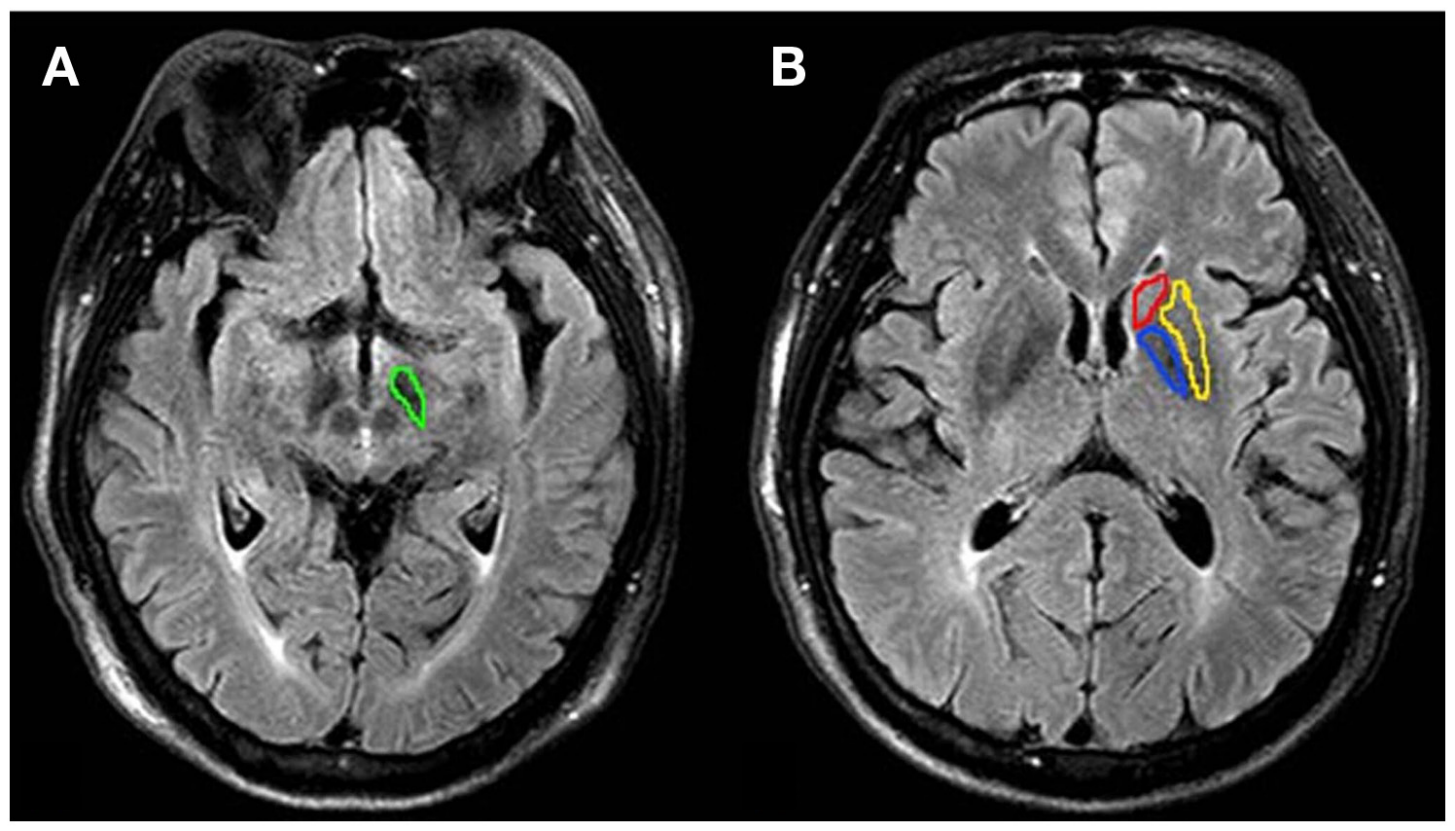
**Examples of the definition of the regions of interest for quantitative analysis**. **(A)** substantia nigra (green), **(B)** globus pallidus (blue), putamen (yellow) and caudate (red). MTRasym(3.5 ppm) and ${\text{MTR}}_{\text{asym}}^{\text{total}}$ were measured for each region. The values of each side were recorded as separate samples.

The quantitative image analysis was performed by two radiologists (CL and RW, who had 5 and 10 years of experience in brain imaging, respectively). The FLAIR images were used as the anatomical reference to draw regions of interest (ROIs; substantia nigra, globus pallidus, putamen, and caudate of both hemispheres) (Figure 1). MTR_asym_(3.5 ppm) and ${\text{MTR}}_{\text{asym}}^{\text{total}}$ were measured for each region. The values of each side were recorded as a separate sample.

#### Diffusion-Tensor Imaging

Imaging analysis was carried out using FSL 4.0 software package (http://www.fmrib.ox.ac.uk/fsl). The first preprocessing step was to correct the motion effect and image distortion due to the eddy current. Next, skull stripping with the brain extraction tool (BET) was applied and brain masks were generated. Third, tensors were determined using DTIFIT, producing FA and MD maps.

**Figure 2:**
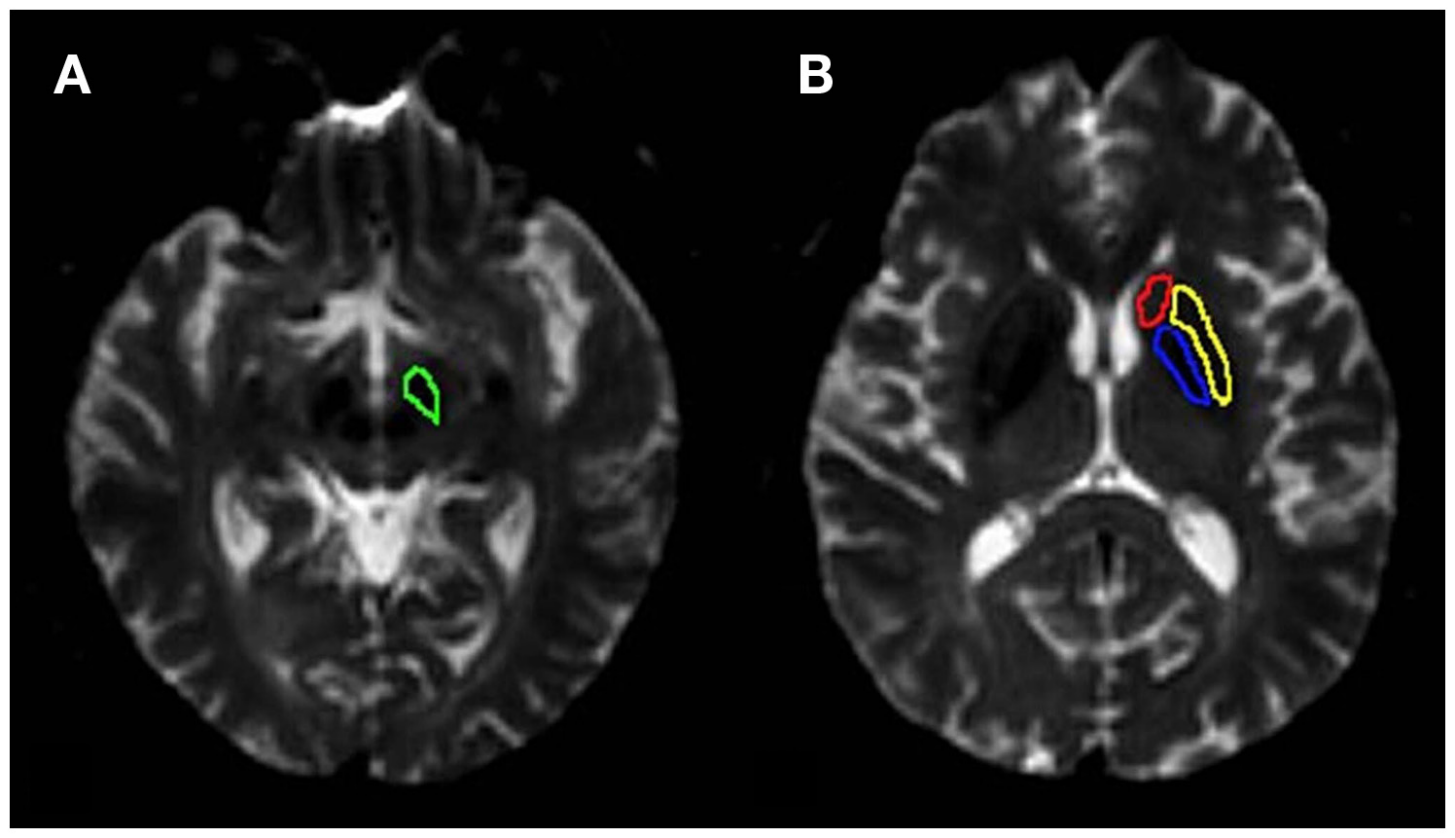
**Examples of the definition of the regions of interest for quantitative analysis**. **(A)** substantia nigra (green), **(B)** globus pallidus (blue), putamen (yellow) and caudate (red). FA and MD were measured for each region. The values of each side were recorded as separate samples.

We used ROI-based analysis in this study. The quantitative image analysis was performed by two radiologists (CL and RW), the same as CEST/APT imaging analysis. The *b* = 0 images were used as the anatomical reference to draw regions of interest (substantia nigra, globus pallidus, putamen, and caudate of both hemispheres) (Figure 2). FA and MD values were measured for each region. The values of each side were recorded as a separate sample. We covered the regions we concerned as much as possible, just the same as we do in CEST/APT analysis, to ensure the ROIs in CEST/APT and DTI were identical as much as possible.

### Statistical Analysis

All data were analyzed using the statistical package SPSS16.0. The average CEST imaging intensities [MTR_asym_(3.5 ppm) and ${\text{MTR}}_{\text{asym}}^{\text{total}}$] and corresponding 95% confidence intervals were calculated for each region, as well as MD and FA values from DTI. Independent-samples t-tests were used to compare the differences in the parameters of CEST and DTI between PD patients and normal controls. Meanwhile, the PD patients were divided into two groups according to the H&Y stages (early stage: H&Y stages 1 and 2 vs. advanced stage: H&Y stages ≥2.5). The values of the parameters at different stages were also compared with one-way ANOVA. The level of significance was set at *P* < 0.05. We used *post hoc* tests to make multiple comparisons between every two groups. Tests of homogeneity of variances *P* values were used before the multiple comparisons. Tukey *post hoc* tests would be used with *P* ≥ 0.05, while Games-howell *post hoc* tests would be used with *P* < 0.05.

## Results

### Substantia Nigra

**Figure 3:**
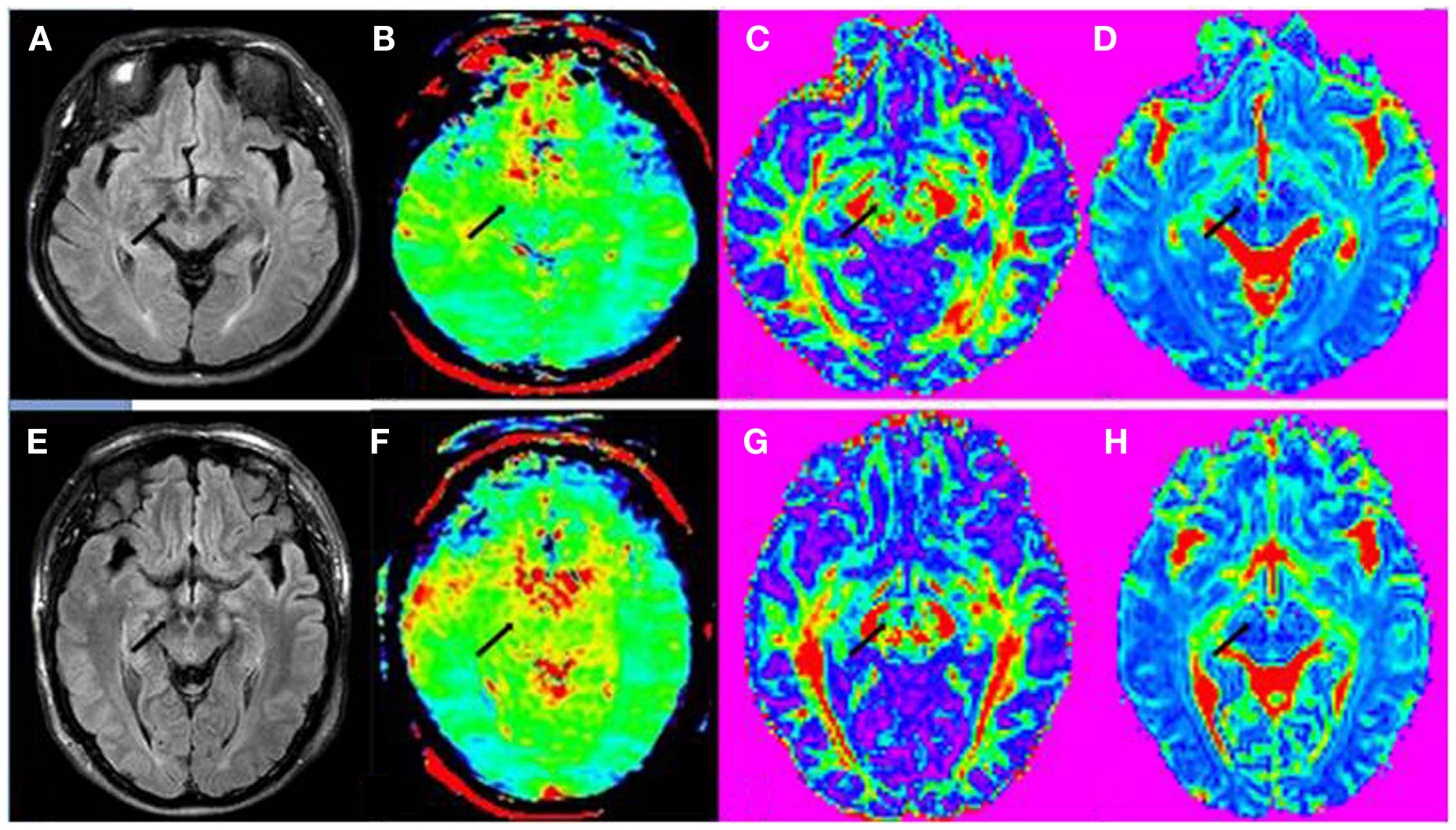
**(A)** FLAIR image, **(B)** APT-weighted image, **(C)** FA image, and **(D)** MD image of a PD patient (female; 53 years old; H&Y stage 3). **(E)** FLAIR image, **(F)** APT-weighted image, **(G)** FA image, and **(H)** MD image of a normal control (male; 65 years old). The CEST/APT imaging acquisition protocol provided B_0_ inhomogeneity-corrected, APT-weighted images with sufficient signal-to-noise ratios. The APT-weighted intensities in regions of the substantia nigra (black arrow) were lower in PD patient than in normal control. The FA values in regions of the substantia nigra (black arrow) seemed to be similar for this PD patient and normal control though they have group differences. The MD value in regions of the substantia nigra (black arrow) also seemed to be similar for this PD patient and normal control.

Table 1 shows the comparison of CEST/APT and DTI values in the substantia nigra for PD patients and for normal controls. The MTR_asym_(3.5 ppm) and ${\text{MTR}}_{\text{asym}}^{\text{total}}$ values from CEST/APT, as well as the FA values from DTI, all showed significant differences on the substantia nigra in PD patients, compared with normal controls. Figure 3 shows the example images in regions of the substantia nigra of a PD patient and a normal control. The value difference between PD patient and normal control can be obviously seen in regions of the substantia nigra (black arrow) in APT-weighted image. However, the FA value difference between PD patient and normal control seemed to be unapparent in the substantia nigra (black arrow) though they have group differences.

**Table 1:** **Comparisons of CEST/APT and DTI parameters values in the substantia nigra for Parkinson’s disease (PD) patients and normal controls (mean **±** 95% CI)**.

		Normal (*n* **=** 23)	PD (*n* **=** 23)	*P* values
CEST/APT	MTR_asym_(3.5 ppm)	1.25 ± 0.18	0.89 ± 0.15	**0.003**
	${\text{MTR}}_{\text{asym}}^{\text{total}}$	3.45 ± 0.44	2.45 ± 0.50	**0.004**
DTI	FA	0.36 ± 0.02	0.32 ± 0.02	<**0.001**
	MD	0.68 ± 0.02	0.70 ± 0.03	0.400

*MTR_asym_(3.5 ppm) was quantified by % water signal intensity. ${\text{MTR}}_{\text{asym}}^{\text{total}}$ was defined as the integral of the MTR_asym_ spectrum in the range of 0–4 ppm. FA, fractional anisotropy; MD, mean diffusivity. Bold indicates significant change*.

Table 2 compares the CEST/APT and DTI parameters values in the substantia nigra for normal controls and for early-stage and advanced-stage PD patients. The MTR_asym_(3.5 ppm) value, the ${\text{MTR}}_{\text{asym}}^{\text{total}}$ value and FA value all showed significant differences between normal controls and advanced-stage PD. The MTR_asym_(3.5 ppm) value of the substantia nigra even decreased significantly from the normal controls to the early-stage PD (*P* = 0.032). A progressive decrease can be seen from normal controls, early-stage PD patients to late-stage PD patients for the MTR_asym_(3.5 ppm) value, the ${\text{MTR}}_{\text{asym}}^{\text{total}}$ value and FA value. No significant differences were found for MD values.

**Table 2:** **CEST/APT and DTI parameters values in the substantia nigra for normal controls and PD patients at different stages**.

		Normal (*n* **=** 23)	Early-stage PD (*n* **=** 12)	Advanced-stage PD (*n* **=** 11)	Tests of homogeneity of variances *P* values	ANOVA			Multiple comparisons *P* values (95% confidence interval)
						df	*F*	*P* values	
CEST/APT	MTR_asym_(3.5 ppm)	1.25 ± 0.18	0.96 ± 0.14	0.81 ± 0.29	**0.035**	2	5.248	**0.007**	**0.032 (0.02, 0.54)**, 0.599 (−0.23, 0.54), **0.032 (0.03, 0.84)**
	${\text{MTR}}_{\text{asym}}^{\text{total}}$	3.45 ± 0.44	2.68 ± 0.50	2.21 ± 0.95	0.097	2	4.980	**0.009**	0.141 (−0.19, 1.73), 0.576 (−0.65, 1.59)**, 0.010 (0.26, 2.23)**
DTI	FA	0.36 ± 0.02	0.34 ± 0.02	0.31 ± 0.03	0.790	2	9.630	**<0.001**	0.149 (−0.01, 0.05), 0.072 (−0.01, 0.07), **<0.001 (0.03, 0.09)**
	MD	0.68 ± 0.02	0.69 ± 0.03	0.70 ± 0.05	**0.003**	2	0.429	0.653	0.837 (−0.06, 0.04), 0.943 (−0.09, 0.07), 0.750 (−0.10, 0.05)

*MTR_asym_(3.5 ppm) was quantified by % water signal intensity. ${\text{MTR}}_{\text{asym}}^{\text{total}}$ was defined as the integral of the MTR_asym_ spectrum in the range of 0–4 ppm. FA, fractional anisotropy; MD, mean diffusivity. Three *P* values in multiple comparisons corresponded to the results between normal controls and early-stage PD patients, between early-stage and advanced-stage PD patients, as well as between normal controls and advanced-stage PD patients, respectively. Bold indicates significant change*.

### Striatum (Globus Pallidus, Putamen, and Caudate)

Tables 3–5 show the comparisons of CEST/APT and DTI parameters values in the globus pallidus, putamen, and caudate for PD patients and normal controls, respectively. The MTR_asym_(3.5 ppm) values of the putamen and caudate were significantly higher in PD patients than in normal controls (*P* = 0.010 and *P* = 0.009, respectively). The MTR_asym_(3.5 ppm) value of the globus pallidus was also higher in PD patients than in normal controls, but the difference was not significant (*P* = 0.125). The ${\text{MTR}}_{\text{asym}}^{\text{total}}$ of the globus pallidus, putamen and caudate were also higher in PD patients than in normal controls, but no significant differences were found (*P* = 0.259, *P* = 0.119, and *P* = 0.573, respectively). No significant differences were found for the FA and MD values of the globus pallidus, putamen, and caudate between PD patients and normal controls.

**Table 3:** **Comparisons of CEST/APT and DTI parameters values in the globus pallidus for Parkinson’s disease (PD) patients and normal controls (mean **±** 95% CI)**.

		Normal (*n* **=** 23)	PD (*n* **=** 23)	*P* values
CEST/APT	MTR_asym_(3.5 ppm)	0.68 ± 0.17	0.84 ± 0.12	0.125
	${\text{MTR}}_{\text{asym}}^{\text{total}}$	2.20 ± 0.29	2.48 ± 0.38	0.259
DTI	FA	0.25 ± 0.01	0.24 ± 0.02	0.122
	MD	0.70 ± 0.03	0.72 ± 0.03	0.227

*MTR_asym_(3.5 ppm) was quantified by % water signal intensity. ${\text{MTR}}_{\text{asym}}^{\text{total}}$ was defined as the integral of the MTR_asym_ spectrum in the range of 0–4 ppm. FA, fractional anisotropy; MD, mean diffusivity*.

**Table 4:** **Comparisons of CEST/APT and DTI parameters values in the putamen for Parkinson’s disease (PD) patients and normal controls (mean **±** 95% CI)**.

		Normal (*n* **=** 23)	PD (*n* **=** 23)	*P* values
CEST/APT	MTR_asym_(3.5 ppm)	0.83 ± 0.13	1.06 ± 0.12	**0.010**
	${\text{MTR}}_{\text{asym}}^{\text{total}}$	2.68 ± 0.25	3.01 ± 0.34	0.119
DTI	FA	0.14 ± 0.01	0.14 ± 0.01	0.520
	MD	0.72 ± 0.01	0.73 ± 0.01	0.142

*MTR_asym_(3.5 ppm) was quantified by % water signal intensity. ${\text{MTR}}_{\text{asym}}^{\text{total}}$ was defined as the integral of the MTR_asym_ spectrum in the range of 0–4 ppm. FA, fractional anisotropy; MD, mean diffusivity. Bold indicates significant change*.

**Table 5:** **Comparisons of CEST/APT and DTI parameters values in the caudate for Parkinson’s disease (PD) patients and normal controls (mean **±** 95% CI)**.

		Normal (*n* **=** 23)	PD (*n* **=** 23)	*P* values
CEST/APT	MTR_asym_(3.5 ppm)	0.84 ± 0.17	1.14 ± 0.14	**0.009**
	${\text{MTR}}_{\text{asym}}^{\text{total}}$	2.79 ± 0.21	2.90 ± 0.35	0.573
DTI	FA	0.16 ± 0.01	0.15 ± 0.01	0.563
	MD	0.74 ± 0.03	0.75 ± 0.01	0.546

*MTR_asym_(3.5 ppm) was quantified by % water signal intensity. ${\text{MTR}}_{\text{asym}}^{\text{total}}$ was defined as the integral of the MTR_asym_ spectrum in the range of 0–4 ppm. FA, fractional anisotropy; MD, mean diffusivity. Bold indicates significant change*.

**Figure 4:**
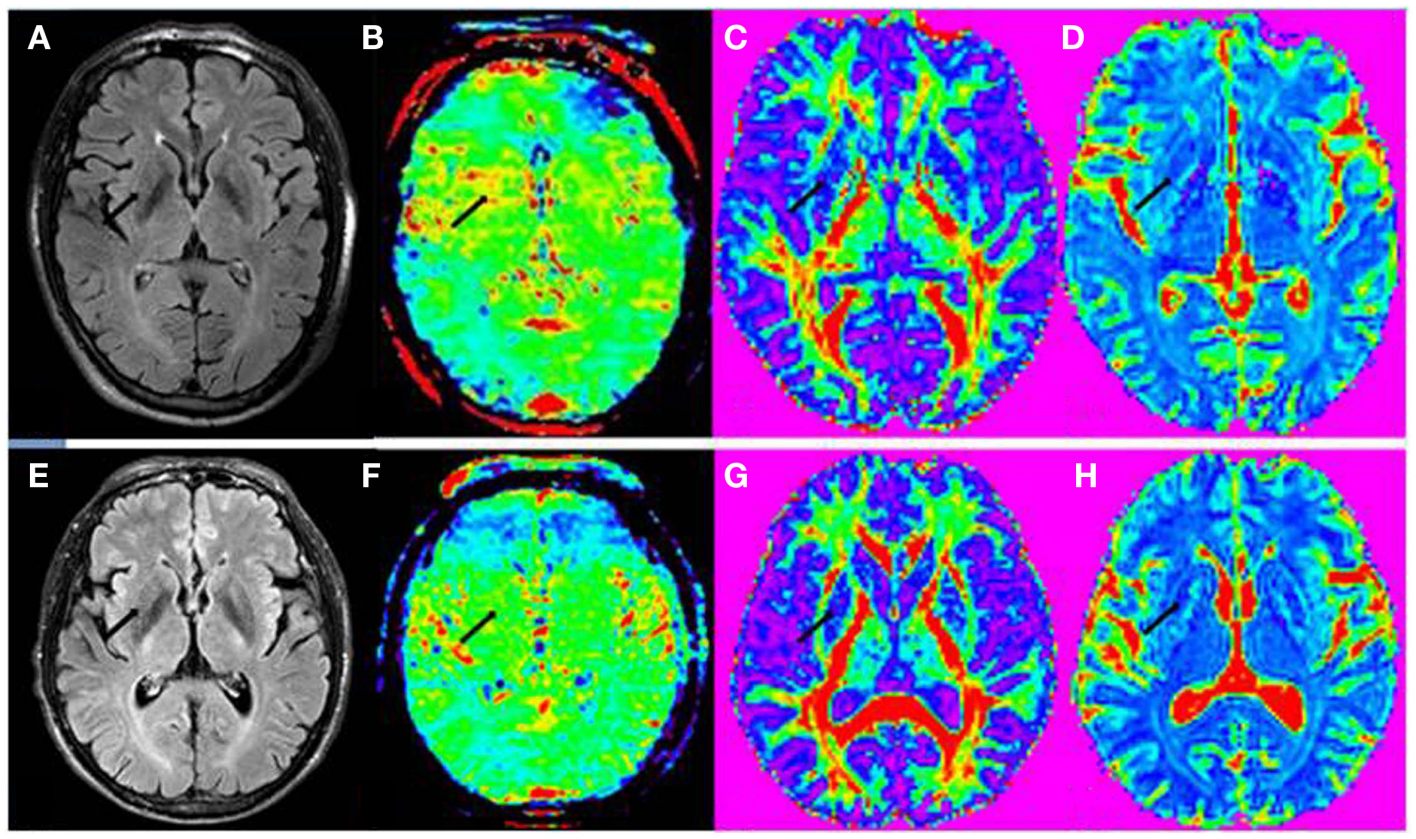
**(A)** FLAIR image, **(B)** APT-weighted image, **(C)** FA image, and **(D)** MD image of a PD patient (male; 74 years old; H&Y stage 2.5). **(E)** FLAIR image, **(F)** APT-weighted image, **(G)** FA image, and **(H)** MD image of a normal control (male; 61 years old). The CEST/APT imaging acquisition protocol provided B_0_ inhomogeneity-corrected, APT-weighted images with sufficient signal-to-noise ratios. The APT-weighted intensities in regions of the striatum (black arrow) were higher in PD patient than in normal control. The FA and MD values in regions of the striatum (black arrow) seemed to be similar for this PD patient and normal control.

Figure 4 shows the example images in the striatum of a PD patient and a normal control. The value difference between PD patient and normal control can be obviously seen in regions of the striatum (black arrow) in APT-weighted image, while no significant differences were found for the FA and MD images (black arrow).

Tables 6–8 further compare CEST/APT and DTI values in the globus pallidus, putamen, and caudate for normal controls and early-stage and advanced-stage PD patients. It can be seen that the MTR_asym_(3.5 ppm) intensities of the putamen and caudate increased significantly from the normal controls to the early-stage PD (*P* = 0.016 and *P* < 0.001, respectively). The MTR_asym_(3.5 ppm) value of the globus pallidus was also higher in early-stage PD patients than in normal controls, but the difference was not significant (*P* = 0.212). Meanwhile, the MTR_asym_(3.5 ppm) of the caudate for PD patients showed a significant decrease from the early stage to the advanced stage (*P* = 0.013).

**Table 6:** **CEST/APT and DTI parameters values in the globus pallidus for normal controls and PD patients at different stages**.

		Normal (*n* **=** 23)	Early-stage PD (*n* **=** 12)	Advanced-stage PD (*n* **=** 11)	Tests of homogeneity of variances *P* values	ANOVA			Multiple comparisons *P* values (95% confidence interval)
						df	*F*	*P* values	
CEST/APT	MTR_asym_(3.5 ppm)	0.68 ± 0.17	0.89 ± 0.14	0.78 ± 0.20	0.065	2	1.470	0.236	0.212 (−0.51, 0.09), 0.739 (−0.24, 0.45), 0.703 (−0.41, 0.20)
	${\text{MTR}}_{\text{asym}}^{\text{total}}$	2.20 ± 0.29	2.60 ± 0.58	2.34 ± 0.53	0.827	2	0.927	0.400	0.366 (−1.09, 0.30), 0.731 (−0.55, 1.07), 0.889 (−0.85, 0.58)
DTI	FA	0.25 ± 0.01	0.23 ± 0.03	0.24 ± 0.03	0.162	2	1.284	0.282	0.294 (−0.01, 0.06), 0.921 (−0.05, 0.04), 0.565 (−0.02, 0.05)
	MD	0.70 ± 0.03	0.71 ± 0.04	0.73 ± 0.04	0.751	2	1.076	0.345	0.849 (−0.07, 0.04), 0.691 (−0.09, 0.04), 0.312 (−0.10, 0.02)

*MTR_asym_(3.5 ppm) was quantified by % water signal intensity. ${\text{MTR}}_{\text{asym}}^{\text{total}}$ was defined as the integral of the MTR_asym_ spectrum in the range of 0–4 ppm. FA, fractional anisotropy; MD, mean diffusivity. Three *P* values in multiple comparisons corresponded to the results between normal controls and early-stage PD patients, between early-stage and advanced-stage PD patients, as well as between normal controls and advanced-stage PD patients, respectively. Bold indicates significant change*.

**Table 7:** **CEST/APT and DTI parameters values in the putamen for normal controls and PD patients at different stages**.

		Normal (*n* **=** 23)	Early-stage PD (*n* **=** 12)	Advanced-stage PD (*n* **=** 11)	Tests of homogeneity of variances *P* values	ANOVA			Multiple comparisons *P* values (95% confidence interval)
						df	*F*	*P* values	
CEST/APT	MTR_asym_(3.5 ppm)	0.83 ± 0.13	1.12 ± 0.12	0.99 ± 0.20	0.279	2	4.142	**0.019**	**0.016 (**−**0.54**, −**0.05)**, 0.500 (−0.15, 0.43), 0.314 (−0.41, 0.10)
	${\text{MTR}}_{\text{asym}}^{\text{total}}$	2.68 ± 0.25	3.10 ± 0.46	2.92 ± 0.55	**0.038**	2	1.427	0.245	0.237 (−1.05, 0.20), 0.849 (−0.65, 1.03), 0.702 (−0.95, 0.48)
DTI	FA	0.14 ± 0.01	0.14 ± 0.02	0.14 ± 0.01	0.260	2	0.250	0.779	0.766 (−0.02, 0.01), 0.954 (−0.02, 0.02), 0.937 (−0.02, 0.02)
	MD	0.72 ± 0.01	0.72 ± 0.02	0.74 ± 0.02	0.505	2	2.046	0.135	0.889 (−0.03, 0.02), 0.360 (−0.05, 0.01), 0.116 (−0.05, 0.01)

*MTR_asym_(3.5 ppm) was quantified by % water signal intensity. ${\text{MTR}}_{\text{asym}}^{\text{total}}$ was defined as the integral of the MTR_asym_ spectrum in the range of 0–4 ppm. FA, fractional anisotropy; MD, mean diffusivity. Three *P* values in multiple comparisons corresponded to the results between normal controls and early-stage PD patients, between early-stage and advanced-stage PD patients, as well as between normal controls and advanced-stage PD patients, respectively. Bold indicates significant change*.

**Table 8:** **CEST/APT and DTI parameters values in the caudate for normal controls and PD patients at different stages**.

		Normal (*n* **=** 23)	Early-stage PD (*n* **=** 12)	Advanced-stage PD (*n* **=** 11)	Tests of homogeneity of variances *P* values	ANOVA			Multiple comparisons *P* values (95% confidence interval)
						df	*F*	*P* values	
CEST/APT	MTR_asym_(3.5 ppm)	0.84 ± 0.17	1.35 ± 0.14	0.91 ± 0.25	0.246	2	8.084	**0.001**	**<0.001 (**−**0.82**, −**0.20)**, **0.013 (0.08, 0.80)**, 0.859 (−0.39, 0.25)
	${\text{MTR}}_{\text{asym}}^{\text{total}}$	2.79 ± 0.21	3.06 ± 0.48	2.73 ± 0.54	**0.008**	2	0.850	0.431	0.531 (−0.90, 0.35), 0.601 (−0.51, 1.18), 0.974 (−0.63, 0.75)
DTI	FA	0.16 ± 0.01	0.16 ± 0.02	0.15 ± 0.01	0.099	2	0.514	0.600	1.000 (−0.02, 0.02), 0.684 (−0.01, 0.03), 0.604 (−0.01, 0.03)
	MD	0.74 ± 0.03	0.75 ± 0.02	0.74 ± 0.02	0.578	2	0.293	0.746	0.726 (−0.07, 0.04), 0.885 (−0.05, 0.07), 0.977 (−0.06, 0.05)

*MTR_asym_(3.5 ppm) was quantified by % water signal intensity. ${\text{MTR}}_{\text{asym}}^{\text{total}}$ was defined as the integral of the MTR_asym_ spectrum in the range of 0–4 ppm. FA, fractional anisotropy; MD, mean diffusivity. Three *P* values in multiple comparisons corresponded to the results between normal controls and early-stage PD patients, between early-stage and advanced-stage PD patients, as well as between normal controls and advanced-stage PD patients, respectively. Bold indicates significant change*.

No significant differences were found for the ${\text{MTR}}_{\text{asym}}^{\text{total}}$values, FA values, and MD values.

## Discussion

In this study, we used two techniques, CEST/APT and DTI, to evaluate PD. The regions we focused on included the substantia nigra and striatum, for they are the main regions involved in PD. We aimed to compare the changes of these regions in PD by these two techniques and find out which can provide more information in the assessment of PD. To our knowledge, very few previous reports evaluated PD with CEST/APT and the present study was the first time to compare CEST and DTI in PD patients.

Chemical exchange saturation transfer imaging is a novel molecular MRI technique that can detect endogenous, low-concentration chemicals in tissue non-invasively (Cai et al., 2012; Haris et al., 2013; Walker-Samuel et al., 2013; Harston et al., 2015). More specifically, APT imaging (a type of CEST imaging, sensitive to amide protons resonating at 3.5 ppm from water) was designed to detect mobile (cytosolic) proteins and peptides. Previous study has indicated the possibility of using CEST/APT in the diagnosis of PD (Li et al., 2014). DTI based upon the diffusivity of water molecules has typically been used to study white matter tract. Although the concerned regions in this study are not the typical targets for DTI studies, diffusion anisotropy changes in gray matter have been reported in aging (Pfefferbaum et al., 2010), brain maturation (Mukherjee et al., 2002), and other neurological disorders (Bozzali et al., 2002). Thus, analyses have performed on gray matter area changes of PD in some studies and the results seemed to be variable.

### Substantia Nigra Result Analysis

The substantia nigra showed significantly lower MTR_asym_(3.5 ppm) signal intensities and lower total CEST signal intensities in PD patients than in normal controls, which may suggest that the changes could be attributed to the loss of dopaminergic neurons or to the depletion of some chemicals with fast exchange protons, such as dopamine. This result is similar to the previous study (Li et al., 2014). FA values of the substantia nigra showed significant difference in PD patients, which may result from the loss of dopaminergic neurons and injury of axons. This result agreed with some publications (Vaillancourt et al., 2009; Du et al., 2012; Skorpil et al., 2012), but in contrast to some other reports showing small or no PD induced decreased FA (Menke et al., 2010; Focke et al., 2011; Kim et al., 2013; Aquino et al., 2014), or even increased nigral FA in PD patients (Wang et al., 2011). For MD values of the substantia nigra, no significant changes were found in PD patients compared to normal controls, though they seemed to have an increase tendency in PD patients. This agreed with some previous reports (Karagulle Kendi et al., 2008; Du et al., 2012; Aquino et al., 2014) but disagreed with some other reports (Gattellaro et al., 2009; Prakash et al., 2012), which found increased nigral MD values. Potential factors contributing to this study heterogeneity may be technical aspects resulting limited quality of FA or MD measurements and different study population characteristics, such as disease severity, as Stefan said in their meta-analysis (Schwarz et al., 2013). From our results, both CEST/APT and DTI parameters are able to differentiate PD from normal controls.

We further divided PD patients into two groups according to the H&Y stages (early stage: H&Y stages 1 and 2 vs. advanced stage: H&Y stages ≥2.5) to find out the CEST/APT and DTI changes in the substantia nigra in different stages of PD. The CEST intensity had a tendency to decrease gradually with the PD progression, no matter the MTR_asym_(3.5 ppm) signal intensities or the total CEST signal intensities. Meanwhile, the FA value also had similar decreased tendency in the progression of PD. As the FA value decrease in PD has been contributed to the continuous dopaminergic neurons loss, such results may provide us indirect evidence that CEST/APT can reveal the loss of dopaminergic neurons in PD (Kirik et al., 2002; Hodaie et al., 2007). However, the CEST/APT seemed to be superior to DTI in the further comparison of different groups. Significant differences were found for both MTR_asym_(3.5 ppm) and total CEST signal intensities between normal controls and advanced-stage PD. Moreover, the MTR_asym_(3.5 ppm) signal intensities of the substantia nigra has decreased significantly in PD patients even in early stage, which indicated the potential of CEST imaging to detect PD patients in early stage. In the other hand, FA values only showed significant differences between normal controls and advanced-stage PD and no significant differences were found for MD although they seemed to increase gradually in PD progression.

### Striatum (Globus Pallidus, Putamen, and Caudate) Result Analysis

For CEST/APT, the MTR_asym_(3.5 ppm) signal intensities and total CEST signal intensities of the globus pallidus, putamen, and caudate were higher in PD patients than in normal controls though the changes in some regions were not significant. This may be associated with increased cytosolic proteins and peptides, as expected (Braak et al., 1999; Tong et al., 2010). When dividing PD patients into early stage and advanced stage, the CEST intensity changes for the globus pallidus, putamen, and caudate were more complicated. It seemed that both the MTR_asym_(3.5 ppm) signal intensities and the total CEST signal intensities reached to the peak at early stage and later dropped at advanced stage. For the putamen and caudate, the MTR_asym_(3.5 ppm) signal intensities even showed significant increases in early stage. This result is similar to the previous study (Li et al., 2014).This indicated CEST intensities in these three regions may play a more important part in the early diagnosis of PD, even though we are not sure the exact mechanism of their changes now. We can only presume the CEST intensities increased in early stage due to the increased of abnormal cytoplasmic proteins (such as α-synuclein) (Braak et al., 1999; Tong et al., 2010) and their decreases in advanced stage may associate with the neurons loss with PD progression or the treatment effect.

Compared with CEST/APT, DTI results seemed not promising. No significant differences were found between PD patients and normal controls in these regions, no matter MD or FA. Even when we further divided PD patients into two groups (early stage and advanced stage) to compare the FA and MD values of three groups, no significant differences were found for every two groups comparisons. Such results accorded with previous reports (Nicoletti et al., 2006; Gattellaro et al., 2009; Focke et al., 2011). In contrast, some studies got the result that these regions may have higher MD values (Kim et al., 2013) or lower FA values (Zhan et al., 2012) in PD. The reason for this inconsistency is not clear. However, we presume that the difference of PD population, scan protocol or the method of postprocessing including thresholding of FA and setting of significance level may be the potential factors.

### Comparison of CEST/APT Imaging and DTI

From our results, CEST/APT imaging had provided more information than DTI in the differentiation of PD. Multiple regions revealed significant changes in the four regions in PD compared to normal controls by the parameters of CEST/APT imaging, while DTI only suggested the FA changes in the substantia nigra. Meanwhile, multiple regions, including the substantia nigra, putamen, and caudate, showed significant MTR_asym_(3.5 ppm) changes even in early stage of PD, which indicated their potential in the early diagnosis of PD. Moreover, the MTR_asym_(3.5 ppm) values of the caudate showed significant changes from early stage to advanced stage of PD, supplying information in the evaluation of PD progression. The FA values of the substantia nigra also decreased significantly from normal controls to advanced-stage PD, but no other significant changes were found for DTI neither in the comparison of normal controls and early-stage PD, nor the comparison of early stage and advanced stage. Though the results may be influenced by the patient population, scan protocol or postprocessing, which may cause the underestimation of the DTI utility in PD evaluation in this study, our preliminary results indeed revealed the superior utility of CEST/APT imaging in the evaluation of PD.

There were some limitations to this study. First, owing to the limitation of the single-slice acquisition protocol for CEST/APT imaging, only two slices were acquired in this study. Thus, we can evaluate the MRI signal changes in these two slices and other regions could not be included in the present study. This shortage may be overcome by 3D imaging acquisition sequence in the future, which has been reported (Zhou et al., 2013b). Second, the ROIs for CEST/APT imaging and DTI were selected respectively, which may cause mismatch of the ROIs selection. Besides, the limited resolution of CEST/APT imaging brought some difficulty for us to select these four small regions exactly for the quantitative analysis. This may influence the comparison result to some extent. Third, the sample number in the present study was relatively small and further studies with large sample analysis are needed to confirm these results.

In conclusion, CEST MR imaging provided multiple CEST signal differences in the regions of substantia nigra and striatum in PD and may be superior to DTI in the diagnosis and severity evaluation of PD. CEST MR imaging has the potential to differentiate PD patients in the early stage and to evaluate the progression of PD. In the coming work, more cases should be collected to confirm the utility of CEST MR imaging. Also, the mechanisms underlying such results should be studied.

## Conflict of Interest Statement

The authors declare that the research was conducted in the absence of any commercial or financial relationships that could be construed as a potential conflict of interest.

## References

[B1] Aquino D. Contarino V. Albanese A. Minati L. Farina L. Grisoli M. (2014). Substantia nigra in Parkinson’s disease: a multimodal MRI comparison between early and advanced stages of the disease. Neurol. Sci. 35, 753–758. 10.1007/s10072-013-1595-2 24337946

[B2] Bozzali M. Cercignani M. Sormani M. P. Comi G. Filippi M. (2002). Quantification of brain gray matter damage in different MS phenotypes by use of diffusion tensor MR imaging. AJNR Am. J. Neuroradiol. 23, 985–988. 12063230 PMC7976912

[B3] Braak H. Sandmann-Keil D. Gai W. Braak E. (1999). Extensive axonal Lewy neurites in Parkinson’s disease: a novel pathological feature revealed by alpha-synuclein immunocytochemistry. Neurosci. Lett. 265, 67–69. 10.1016/S0304-3940(99)00208-6 10327208

[B4] Cai K. J. Haris M. Singh A. Kogan F. Greenberg J. H. Hariharan H. (2012). Magnetic resonance imaging of glutamate. Nat. Med. 18, 302–306. 10.1038/nm.2615 22270722 PMC3274604

[B5] Cnyrim C. D. Kupsch A. Ebersbach G. Hoffmann K. T. (2014). Diffusion tensor imaging in idiopathic Parkinson’s disease and multisystem atrophy (Parkinsonian type). Neurodegener. Dis. 13, 1–8. 10.1159/000348512 23711586

[B6] Dauer W. Przedborski S. (2003). Parkinson’s disease: mechanisms and models. Neuron 39, 889–909. 10.1016/S0896-6273(03)00568-3 12971891

[B7] Du G. Lewis M. M. Sen S. Wang J. Shaffer M. L. Styner M. (2012). Imaging nigral pathology and clinical progression in Parkinson’s disease. Mov. Disord. 27, 1636–1643. 10.1002/mds.25182 23008179 PMC3510346

[B8] Focke N. K. Helms G. Pantel P. M. Scheewe S. Knauth M. Bachmann C. G. (2011). Differentiation of typical and atypical Parkinson syndromes by quantitative MR imaging. AJNR Am. J. Neuroradiol. 32, 2087–2092. 10.3174/ajnr.A2865 21998102 PMC7964420

[B9] Gattellaro G. Minati L. Grisoli M. Mariani C. Carella F. Osio M. (2009). White matter involvement in idiopathic Parkinson disease: a diffusion tensor imaging study. AJNR Am. J. Neuroradiol. 30, 1222–1226. 10.3174/ajnr.A1556 19342541 PMC7051338

[B10] Hagmann P. Jonasson L. Maeder P. Thiran J. P. Wedeen V. J. Meuli R. (2006). Understanding diffusion MR imaging techniques: from scalar diffusion-weighted imaging to diffusion tensor imaging and beyond. Radiographics 26, S205–S223. 10.1148/rg.26si065510 17050517

[B11] Haris M. Nath K. Cai K. J. Singh A. Crescenzi R. Kogan F. (2013). Imaging of glutamate neurotransmitter alterations in Alzheimer’s disease. NMR. Biomed. 26, 386–391. 10.1002/nbm.2875 23045158 PMC3556355

[B12] Harston G. W. Tee Y. K. Blockley N. Okell T. W. Thandeswaran S. Shaya G. (2015). Identifying the ischaemic penumbra using pH-weighted magnetic resonance imaging. Brain 138, 36–42. 10.1093/brain/awu374 25564491 PMC4285197

[B13] Henkelman R. M. Stanisz G. J. Graham S. J. (2001). Magnetization transfer in MRI: a review. NMR. Biomed. 14, 57–64. 10.1002/nbm.683 11320533

[B14] Heo H.-Y. Zhang Y. Lee D.-H. Hong X. Zhou J. (2015). Quantitative assessment of amide proton transfer (APT) and nuclear Overhauser enhancement (NOE) imaging with extrapolated semi-solid magnetization transfer reference (EMR) signals: application to a rat glioma model at 4.7 T. Magn. Reson. Med. 10.1002/mrm.25795 PMC4561043 25753614

[B15] Hodaie M. Neimat J. S. Lozano A. M. (2007). The dopaminergic nigrostriatal system and Parkinson’s disease: molecular events in development, disease, and cell death, and new therapeutic strategies. Neurosurgery 60, 17–28. discussion 28–30 17228250 10.1227/01.NEU.0000249209.11967.CB

[B16] Hughes A. J. Daniel S. E. Kilford L. Lees A. J. (1992). Accuracy of clinical diagnosis of idiopathic Parkinson’s disease: a clinico-pathological study of 100 cases. J. Neurol. Neurosurg. Psychiatr. 55, 181–184. 10.1136/jnnp.55.3.181 1564476 PMC1014720

[B17] Hughes A. J. Daniel S. E. Lees A. J. (2001). Improved accuracy of clinical diagnosis of Lewy body Parkinson’s disease. Neurology 57, 1497–1499. 10.1212/WNL.57.8.1497 11673599

[B18] Kamagata K. Tomiyama H. Hatano T. Motoi Y. Abe O. Shimoji K. (2014). A preliminary diffusional kurtosis imaging study of Parkinson disease: comparison with conventional diffusion tensor imaging. Neuroradiology 56, 251–258. 10.1007/s00234-014-1327-1 24468858

[B19] Karagulle Kendi A. T. Lehericy S. Luciana M. Ugurbil K. Tuite P. (2008). Altered diffusion in the frontal lobe in Parkinson disease. AJNR Am. J. Neuroradiol. 29, 501–505. 10.3174/ajnr.A0850 18202242 PMC8118887

[B20] Kim H. J. Kim S. J. Kim H. S. Choi C. G. Kim N. Han S. (2013). Alterations of mean diffusivity in brain white matter and deep gray matter in Parkinson’s disease. Neurosci. Lett. 550, 64–68. 10.1016/j.neulet.2013.06.050 23831353

[B21] Kirik D. Rosenblad C. Burger C. Lundberg C. Johansen T. E. Muzyczka N. (2002). Parkinson-like neurodegeneration induced by targeted overexpression of alpha-synuclein in the nigrostriatal system. J. Neurosci. 22, 2780–2791. 11923443 10.1523/JNEUROSCI.22-07-02780.2002 PMC6758323

[B22] Kogan F. Hariharan H. Reddy R. (2013). Chemical exchange saturation transfer (CEST) imaging: description of technique and potential clinical applications. Curr. Radiol. Rep. 1, 102–114. 10.1007/s40134-013-0010-3 23730540 PMC3665411

[B23] Lenfeldt N. Hansson W. Larsson A. Nyberg L. Birgander R. Forsgren L. (2013). Diffusion tensor imaging and correlations to Parkinson rating scales. J. Neurol. 260, 2823–2830. 10.1007/s00415-013-7080-2 23974647

[B24] Li C. Peng S. Wang R. Chen H. Su W. Zhao X. (2014). Chemical exchange saturation transfer MR imaging of Parkinson’s disease at 3 Tesla. Eur. Radiol. 24, 2631–2639. 10.1007/s00330-014-3241-7 25038850 PMC4471479

[B25] Menke R. A. Jbabdi S. Miller K. L. Matthews P. M. Zarei M. (2010). Connectivity-based segmentation of the substantia nigra in human and its implications in Parkinson’s disease. Neuroimage 52, 1175–1180. 10.1016/j.neuroimage.2010.05.086 20677376

[B26] Mukherjee P. Miller J. H. Shimony J. S. Philip J. V. Nehra D. Snyder A. Z. (2002). Diffusion-tensor MR imaging of gray and white matter development during normal human brain maturation. AJNR Am. J. Neuroradiol. 23, 1445–1456. 12372731 PMC7976805

[B27] Nicoletti G. Lodi R. Condino F. Tonon C. Fera F. Malucelli E. (2006). Apparent diffusion coefficient measurements of the middle cerebellar peduncle differentiate the Parkinson variant of MSA from Parkinson’s disease and progressive supranuclear palsy. Brain 129, 2679–2687. 10.1093/brain/awl166 16815875

[B28] Olanow C. W. Rascol O. Hauser R. Feigin P. D. Jankovic J. Lang A. (2009). A double-blind, delayed-start trial of rasagiline in Parkinson’s disease. N. Engl. J. Med. 361, 1268–1278. 10.1056/NEJMoa0809335 19776408

[B29] Pfefferbaum A. Adalsteinsson E. Rohlfing T. Sullivan E. V. (2010). Diffusion tensor imaging of deep gray matter brain structures: effects of age and iron concentration. Neurobiol. Aging 31, 482–493. 10.1016/j.neurobiolaging.2008.04.013 18513834 PMC2815127

[B30] Prakash B. D. Sitoh Y. Y. Tan L. C. Au W. L. (2012). Asymmetrical diffusion tensor imaging indices of the rostral substantia nigra in Parkinson’s disease. Parkinsonism Relat. Disord. 18, 1029–1033. 10.1016/j.parkreldis.2012.05.021 22705126

[B31] Schwarz S. T. Abaei M. Gontu V. Morgan P. S. Bajaj N. Auer D. P. (2013). Diffusion tensor imaging of nigral degeneration in Parkinson’s disease: a region-of-interest and voxel-based study at 3 T and systematic review with meta-analysis. Neuroimage Clin. 3, 481–488. 10.1016/j.nicl.2013.10.006 24273730 PMC3830065

[B32] Skorpil M. Soderlund V. Sundin A. Svenningsson P. (2012). MRI diffusion in Parkinson’s disease: using the technique’s inherent directional information to study the olfactory bulb and substantia nigra. J. Parkinsons Dis. 2, 171–180. 10.3233/JPD-2012-12091 23939442

[B33] Tong J. Wong H. Guttman M. Ang L. C. Forno L. S. Shimadzu M. (2010). Brain alpha-synuclein accumulation in multiple system atrophy, Parkinson’s disease and progressive supranuclear palsy: a comparative investigation. Brain 133, 172–188. 10.1093/brain/awp282 19903734

[B34] Vaillancourt D. E. Spraker M. B. Prodoehl J. Abraham I. Corcos D. M. Zhou X. J. (2009). High-resolution diffusion tensor imaging in the substantia nigra of de novo Parkinson disease. Neurology 72, 1378–1384. 10.1212/01.wnl.0000340982.01727.6e 19129507 PMC2677508

[B35] van Buchem M. A. Tofts P. S. (2000). Magnetization transfer imaging. Neuroimaging Clin. N. Am. 10, 771–788. 11359724

[B36] Vinogradov E. Sherry A. D. Lenkinski R. E. (2013). CEST: from basic principles to applications, challenges and opportunities. J. Magn. Reson. 229, 155–172. 10.1016/j.jmr.2012.11.024 23273841 PMC3602140

[B37] Walker-Samuel S. Ramasawmy R. Torrealdea F. Rega M. Rajkumar V. Johnson S. P. (2013). In vivo imaging of glucose uptake and metabolism in tumors. Nat. Med. 19, 1067–1072. 10.1038/nm.3252 23832090 PMC5275770

[B38] Wang J. J. Lin W. Y. Lu C. S. Weng Y. H. Ng S. H. Wang C. H. (2011). Parkinson disease: diagnostic utility of diffusion kurtosis imaging. Radiology 261, 210–217. 10.1148/radiol.11102277 21771952

[B39] Zhan W. Kang G. A. Glass G. A. Zhang Y. Shirley C. Millin R. (2012). Regional alterations of brain microstructure in Parkinson’s disease using diffusion tensor imaging. Mov. Disord. 27, 90–97. 10.1002/mds.23917 21850668 PMC4472452

[B40] Zhou J. Blakeley J. O. Hua J. Kim M. Laterra J. Pomper M. G. (2008). Practical data acquisition method for human brain tumor amide proton transfer (APT) imaging. Magn. Reson. Med. 60, 842–849. 10.1002/mrm.21712 18816868 PMC2579754

[B41] Zhou J. Hong X. Zhao X. Gao J.-H. Yuan J. (2013a). APT-weighted and NOE-weighted image contrasts in glioma with different RF saturation powers based on magnetization transfer ratio asymmetry analyses. Magn. Reson. Med. 70, 320–327. 10.1002/mrm.24784 23661598 PMC3723702

[B42] Zhou J. Zhu H. Lim M. Blair L. Quinones-Hinojosa A. Messina S. A. (2013b). Three-dimensional amide proton transfer MR imaging of gliomas: initial experience and comparison with gadolinium enhancement. J. Magn. Reson. Imaging 38, 1119–1128. 10.1002/jmri.24067 23440878 PMC3664658

[B43] Zhou J. Payen J. Wilson D. A. Traystman R. J. van Zijl P. C. M. (2003). Using the amide proton signals of intracellular proteins and peptides to detect pH effects in MRI. Nat. Med. 9, 1085–1090. 10.1038/nm907 12872167

[B44] Zhou J. van Zijl P. C. (2006). Chemical exchange saturation transfer imaging and spectroscopy. Prog. Nucl. Magn. Reson. Spectrosc. 48, 109–136. 10.1016/j.pnmrs.2006.01.001 33198968

